# Association between physical and psychosocial demands and musculoskeletal pain in health workers

**DOI:** 10.47626/1679-4435-2022-765

**Published:** 2023-02-13

**Authors:** Giselle de Santana Vilasboas Dantas, Jefferson Paixão Cardoso, Tânia Maria de Araújo

**Affiliations:** 1 Núcleo de Estudos em Saúde da População, Universidade Estadual do Sudoeste da Bahia, Jequié, BA, Brazil; 2 Núcleo de Epidemiologia, Universidade Estadual de Feira de Santana, Feira de Santana, BA, Brazil

**Keywords:** occupational health, health care workers, working conditions, musculoskeletal pain, saúde do trabalhador, pessoal de saúde, condições de trabalho, dor musculoesquelética

## Abstract

**Introduction:**

The physical and psychosocial demands of work are important factors in the genesis of musculoskeletal pain. Identification of these dimensions and their interfaces with workers’ individual characteristics could improve understanding of these outcomes.

**Objectives:**

To analyze the associations between the physical and psychosocial demands of work and occurrence of musculoskeletal pain in health care workers.

**Methods:**

This was a cross-sectional study conducted with health care workers. The exposure variables were psychosocial aspects and physical demands, investigated using the Job Content Questionnaire, and the outcomes were musculoskeletal pain in lower limbs, upper limbs, and the back, investigated as self-report of pain. A multivariate analysis was conducted to investigate associations between exposures and outcomes.

**Results:**

The factors associated with musculoskeletal pain in the three areas of the body studied were female sex, physical inactivity, and “poor” self-rated health status. Additionally, being a contract worker was associated with musculoskeletal pain in the lower limbs and back. Not participating in leisure activities and being responsible for direct provision of health care were associated with pain in the lower limbs. Being the person responsible for the housework and doing the housework were associated with pain in upper limbs. Differences between the demands of tasks, poor availability of the technical resources to perform activities, and absence of leisure activities were associated with back pain.

**Conclusions:**

It was concluded that both physical demands and psychosocial demands are associated with musculoskeletal pain in health care workers.

## Abstract (pt)

**Introdução:**

As demandas físicas e psicossociais do trabalho constituem importantes fatores para a ocorrência de dor musculoesquelética. Conhecer essas dimensões e suas interfaces com as características individuais do trabalhador pode ajudar a compreender a ocorrência desses desfechos.

**Objetivos:**

Analisar a associação entre demandas físicas e psicossociais do trabalho e a ocorrência de dor musculoesquelética em trabalhadores da saúde.

**Métodos:**

Tratou-se de um estudo de corte transversal com trabalhadores da saúde. As variáveis de exposição foram os aspectos psicossociais e as demandas físicas investigadas por meio do Job Content Questionnaire, e o desfecho foi a dor musculoesquelética em membros inferiores, superiores e na região dorsal, investigada pela queixa de dor autorreferida. A análise multivariável foi utilizada para investigar a associação entre as exposições e os desfechos.

**Resultados:**

Os fatores associados a dor musculoesquelética nos três segmentos corporais foram ser do sexo feminino, não praticar atividade física e perceber o estado de saúde como “ruim”. O vínculo de trabalho “não efetivo” esteve associado a dor musculoesquelética em membros inferiores e na região dorsal. A não participação em atividades de lazer e a prestação de assistência direta à saúde estiveram associadas a dor em membros inferiores. Ser o principal responsável pelas atividades domésticas e realizá-las mostraram-se associados a dor em membros superiores. As diferenças entre as exigências das tarefas, os recursos técnicos disponíveis ruins para a execução das atividades e a ausência de atividades de lazer estiveram associados a dor na região dorsal.

**Conclusões:**

Conclui-se que tanto as demandas físicas quanto as psicossociais estão associadas à dor musculoesquelética em trabalhadores da saúde.

## INTRODUCTION

Sickness caused by employment activities, whether physical or emotional, has increased progressively in many occupational categories. This is true of people who work in health care, whether they are workers who provide care directly or are involved in service provision.^1^

One class of physical conditions that affect workers’ health is musculoskeletal disorders,^2^ which includes inflammation, pain, and degeneration of muscles, tendons, joints, nerves, and cartilage, causing functional limitations. These disorders are caused by excessive use of the musculoskeletal system compounded by a lack of time for recovery^3^ and can result from the action of multiple factors inherent to employment activity, especially the physical and psychosocial demands of work.^4^

In addition to the excessive demand on an individual’s capacity, activities that require use of extreme force can also impair circulation to the muscles, leading to tension and exhaustion and increasing the time needed for recovery after performing the task. Excessive effort combined with little or no time for recovery can provoke pain.^5^

Negative psychosocial aspects of work include monotony, work overload, time pressure, little control over one’s own work, lack of social support, poor relationships with colleagues, lack of autonomy to perform tasks, and disorganized working procedures and these factors can also be related to emergence of musculoskeletal pain (MSP).^6^ This is because of increased muscle tension and a reduction in the workers’ capacity to deal with symptoms, increasing the perception of pain.^6^

Activities performed in inappropriate positions, repetitive and monotonous tasks, with no rest breaks, with inappropriate furniture and equipment, and those involving lifting and carrying loads contribute to MSP, as does work performed under high psychosocial demands, resulting in greater physical effort, excessive working hours, few breaks for rest, and insufficient changes of position.^7^

In addition to the physical and psychosocial demands, personal characteristics such as age and sex, lifestyle habits, comorbidities,^8^ and educational level^9^ can also contribute to occurrence of musculoskeletal symptomology.^8^ Although the personal characteristics cannot be changed, individual behavior and the characteristics of work can be changed in order to improve working conditions and, consequently, reduce its impact on workers’ health.^10^

In view of the above, the objective of this study was to analyze the association between the physical and psychosocial demands of work and occurrence of MSP in health care workers.

## METHODS

### STUDY DESIGN

This was a cross-sectional study focused on investigating the association between physical and psychosocial demands and occurrence of MSP in health care workers from six towns in Bahia, Brazil, in 2012. The study is derived from the multicenter project “Working conditions, employment conditions, and health of health care workers in Bahia” (project financed by the Bahia State Research Funding Agency [FAPESB - Fundação de Amparo à Pesquisa do Estado da Bahia]: Research Project for the Unified Health System [SUS - Sistema Único de Saúde] SUS0024/2009 and SUS0022/2014 and the National Council for Scientific and Technological Development [CNPq - Conselho Nacional de Desenvolvimento Científico e Tecnológico]: process No. 480611/2010-6).

### STUDY PARTICIPANTS

The underlying study population comprised workers attached to primary care and medium complexity care services in the towns of Feira de Santana, Santo Antônio de Jesus, Jequié, Salvador, Itabuna, and Itaberaba who were working full time and agreed to participate in the project. A representative sample was selected using a randomization procedure employing sampling stratified by geographic area, level of care complexity, and workers’ occupational categories, calculated using a formula for a population of 6,191 health care workers from the five towns, with a 79.2% vaccination prevalence, 3% error, and 95% confidence interval (95%CI). Based on these parameters, the sample size would be 763 workers. While the study was ongoing, an additional center was included, with a population of 502 workers, raising the total to 6,693 workers. However, a total sample of 3,343 people were interviewed.

The study power was calculated for combined evaluation of psychosocial aspects and MSP. Considering an overall prevalence of MSP of 53.4% among exposed workers (exposed to high physical demand and high psychosocial demand) and a 31% prevalence among unexposed workers (exposed to low physical demand and low psychosocial demand), the study power was 99.9%.

### DATA COLLECTION

Data were collected in 2011 and 2012 using a questionnaire made up of sections containing questions on sociodemographic, occupational, and organizational characteristics of work; lifestyle; and health problems. After contacting the municipal health departments responsible for each participating center and the administrations of each of these health centers, the units were contacted and data collection forms were distributed to the workers who had been selected (the forms were administered to workers with secondary education). The team of interviewers was trained before data collection to standardize behavior and procedures. A pilot study was run in a town in Bahia with 30 health care workers.

### OUTCOME

The study’s outcome variable was MSP, investigated by self-report pain indicated on a Likert response scale (never, rarely, infrequently, frequently, and very frequently). The body areas studied were lower limbs (LL), upper limbs (UL), lower back, and upper back. The last two categories were re-categorized as back pain.

The response options “frequently” or “very frequently” were defined as cases of MSP and the options “infrequently”, “rarely”, and “never” were defined as absence of MSP. This approach has been employed successfully to assess presence of MSP among workers in Brazil.^11,12^

### PRINCIPAL EXPOSURE VARIABLES

The exposure variables were physical and psychosocial demands, investigated using the Job Content Questionnaire (JCQ). This instrument contains questions with Likert response scales (1 = completely disagree, 2 = disagree, 3 = agree, and 4 = completely agree) for psychosocial aspects (control over one’s own job and psychological demands) and physical demands. The responses were used to calculate scores for each of these scales, as described in the JCQ Manual (www.JCQCenter.org). Later, once the estimated scores had been collected, these variables were dichotomized, adopting the median of the distribution of each scale as its cutoff point. Values below the median were classified as low demand and values over the median were defined as high demand. These data were used to define groups as undemanding work (a combination of low demand and high control), passive work (low demand and low control), active work (high demand and high control) and highly demanding work (high demand and low control).^13^

Participants were then allocated to one of two groups: a group with high psychosocial exposure, comprising those who were under high psychological demand, had low control over their work, and had low social support; and a group with low psychosocial exposure, comprising those who were under low psychological demand, had high control over their work, and had high social support.

After this categorization (dichotomization of the variables), workers were classified into groups according to whether they had exposure to physical and psychosocial demands, so that those who were unexposed to psychosocial aspects and unexposed to physical demands were classified as P_00_; those who were unexposed to high physical demand, but exposed to high psychosocial demand were classified as P_01_; those exposed to high physical demand and unexposed to high psychosocial demand were allocated to P_10_; and those who were exposed to both psychosocial aspects and physical demand, were classified as P_11_.^14^

### COVARIATES

The covariates considered for analysis were organized in blocks as follows:

- Block I - Principal exposure: physical demand and psychosocial demand (combined).
- Block II - Sociodemographics: sex (male; female); age group (19-33 years; 34-43 years; ≥ 44 years); marital status (single; married/stable consensual relationship; widowed/divorced/separated/estranged); educational level (primary education; secondary education/technical college; higher education); race/color (white/yellow; black/brown; indigenous); has children (yes; no).
- Block IIIa - Work performed at the unit: job type (direct health care provision; support staff); time in job in years (up to 4 years; 5-12 years; ≥ 13 years); type of employment (permanent; contract); compatibility of activities with job (yes; no); work shift (day; night; on call); working week (up to 20 hours; 21-39 hours; ≥ 40 hours); has another job (yes; no); condition of chairs and tables, technical resources and equipment; level of demand of tasks; and resources available (the response options for these variables are “good” or “poor”).
- Block IIIb - Housework: being the person responsible for the housework; doing the housework (yes; no).
- Block IV - Health status and lifestyle habits: participation in leisure activities and physical activity (yes; no); self-rated health status (good; poor).

### ANALYSIS OF THE DATA

Workers’ characteristics were analyzed by calculating the frequencies of the covariates in the blocks described above and the combined exposure variable. Exploratory bivariate analyses were then conducted to guide inclusion of variables in the logistic regression model. Variables with p ≤ 0.20 were selected for modeling.

**Figure 1:**
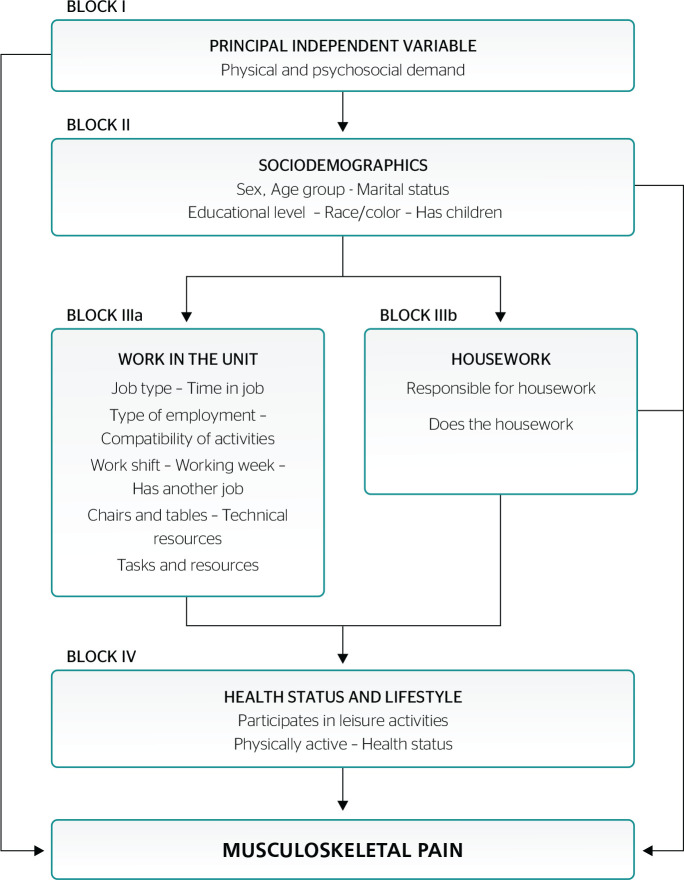
Conceptual model for analysis of factors associated with physical and psychosocial demands and occurrence of musculoskeletal pain.

The association analysis was based on a hierarchical conceptual model (Figure 1), constructed on the basis of the proximal-distal relationships between the variables in the blocks and the outcome. This type of analysis can be used to work with large numbers of variables,^15,16^ which are inserted in stages, starting with the most distal belonging to the same block, simultaneously with the outcome.^16,17^

In the inter-block hierarchical stage, variables with p ≤ 0.05 in the bivariate analyses and variables with theoretical relevance according to the literature were retained in the model. Variables that did not exhibit associations in the intrablock analysis were removed when entering the subsequent block.

### ETHICAL QUESTIONS

The study respected all ethical principles. The project was approved by the research ethics committee at Universidade Estadual de Feira de Santana, protocol 081/2009 and Ethics Appraisal Submission Certificate number 0086.0.059.000.09.

## RESULTS

A total of 3,343 health care workers were interviewed. Of these, 23.2% were not exposed to any of the exposures (high psychosocial or physical demand), 25.5% were only exposed to high psychosocial demand, 16.4%, were only exposed to high physical demand, and 34.9% were exposed both to high physical demand and to high psychosocial demand.

According to Table 1, when other combined exposure categories were compared, it was observed that group P_11_ (high physical demand and high psychosocial demand) had a predominance of female workers (80.3%), those in the age group 19 to 33 years (36.7%), those married/in a stable consensual relationship (57.3%), those with secondary education/technical college (58.9%) and black/brown skin color (81.3%), those who do the housework (93.9%) and are the person responsible for the housework (55.6%), and those who do not participate in leisure activities (20.1%), who are physically inactive (52.7%), and who rate their own health status as poor (5.2%).

**Table 1:** Characteristics of health care workers according to combined exposures: sociodemographic variables, domestic activities, and lifestyle habits, Feira de Santana, Santo Antônio de Jesus, Jequié, Salvador, Itabuna, and Itaberaba, Brazil, 2011 2012

Variables	P^00^		P^01^		P^10^		P^11^	
	n	%	n	%	n	%	n	%
Sex								
Female	548	77.8	590	76.1	396	79.4	851	80.3
Male	156	22.2	185	23.9	103	20.6	209	19.7
Age group (years)								
19-33	226	32.7	279	36.7	152	31.0	382	36.7
34-43	228	32.9	246	32.4	169	34.5	343	33.0
≥ 44	238	34.4	235	30.9	169	34.5	315	30.3
Marital status								
Single	226	32.1	259	33.4	159	31.9	336	31.8
Married/stable consensual relationship	403	57.2	442	57.0	279	56.0	606	57.3
Widowed/divorced/separated/estranged	75	10.7	74	9.6	60	12.1	115	10.9
Educational level								
Up to primary education	25	3.6	23	3.0	23	4.6	44	4.2
Secondary education/technical college	320	46.2	386	50.7	292	58.9	617	58.9
Higher education	348	50.2	353	46.3	181	36.5	387	36.9
Race/color								
White/yellow	127	18.4	128	16.8	84	17.2	184	17.6
Black/brown	553	80.3	622	81.4	398	81.2	849	81.3
Indigenous	9	1.3	14	1.8	8	1.6	11	1.1
Has children								
Yes	469	66.9	521	67.6	357	71.7	732	69.1
No	232	33.1	250	32.4	141	28.3	327	30.9
Is the person responsible for the housework								
Yes	298	43.8	383	50.9	287	58.6	574	55.6
No	382	56.2	369	49.1	203	41.4	459	44.4
Does the housework								
Yes	620	89.5	679	89.7	462	93.5	976	93.9
No	73	10.5	78	10.3	32	6.5	64	6.1
Leisure activities								
Yes	607	87.0	660	85.6	411	82.7	843	79.9
No	91	13.0	111	14.4	86	17.3	212	20.1
Is physically active								
Yes	395	56.7	442	54.9	267	54.5	499	47.3
No	301	43.3	347	45.1	223	45.5	555	52.7
Self-rated health status								
Good	686	98.0	747	97.4	489	98.2	995	94.8
Poor	14	2.0	20	2.6	9	1.8	55	5.2

P_00_ = low physical demand and low psychosocial demand; P_01_ = low physical demand and high psychosocial demand; P_10_ = high physical demand and low psychosocial demand; P_11_ = high physical demand and high psychosocial demand.

With relation to job characteristics and working environment (Table 2), participants in group P_11_ were predominantly support staff (31.5%); those with 13 years or more in the job (27.4%); performing activities compatible with their jobs (1.3%); working on call (17.5%); working ≥ 40 hours per week (72.3%); and those who did not have another job (80.4%). Similarly, workers in P_11_ rated the condition of tables and chairs as poor (44.9%), technical resources and equipment as poor (43.4%) the level of demand of tasks as poor, and the resources available as poor (28.4%). It was also observed that those who always stand up, rarely sit down, or always walk to perform their activities accounted, respectively, for 56.2, 42, and 61.5% of the P_11_ category. Finally, those who always lift, carry, or push loads and have breaks during the work shift were, respectively, 28.9 and 56.5% of P_11_.

**Table 2:** Characteristics of health care workers according to combined exposures: characteristics of the job and workplace, Feira de Santana, Santo Antônio de Jesus, Jequié, Salvador, Itabuna, and Itaberaba, Brazil, 2011-2012

Variables	P^00^		P^01^		P^10^		P^11^	
	n	%	n	%	n	%	n	%
Job type								
Direct health care	489	69.5	525	67.9	358	72.0	724	68.5
Support staff	215	30.5	248	32.1	139	28.0	333	31.5
Time in job (years)								
Up to 4	272	39.1	309	40.4	159	32.0	376	35.6
5-12	239	34.3	264	34.5	211	42.4	391	37.0
≥ 13	185	26.6	192	25.1	127	25.6	290	27.4
Type of employment								
Permanent	427	61.2	499	64.6	348	70.4	702	66.9
Contract	271	38.8	273	35.4	146	29.6	348	33.1
Activities compatible with the job								
Yes	698	99.4	767	99.0	496	99.6	1,045	98.7
No	4	0.6	8	1.0	2	0.4	14	1.3
Work shift								
Day	596	85.4	651	84.7	406	82.4	843	80.0
Night	9	1.3	16	3.0	4	0.8	26	2.5
On call	93	13.3	102	13.3	83	16.8	184	17.5
Working week (hours)								
Up to 20	96	13.7	82	10.6	50	10.1	85	8.0
21-39	161	23.0	177	22.8	91	18.3	209	19.7
≥ 40	444	63.3	516	66.6	356	71.6	766	72.3
Has another job								
Yes	206	29.7	186	24.4	118	24.0	206	19.6
No	488	70.3	577	75.6	374	76.0	847	80.4
Condition of chairs and tables								
Good	375	77.8	356	68.7	192	70.1	360	55.1
Poor	107	22.2	162	31.3	82	29.9	293	44.9
Technical resources and equipment								
Good	514	73.5	489	63.4	344	69.1	596	56.6
Poor	185	26.5	282	36.6	154	30.9	457	43.4
Level of demand of tasks and resources available								
Boas	604	86.4	571	73.9	411	82.9	757	71.6
Poor	95	13.6	202	26.1	85	17.1	300	28.4
Stands up								
Rarely	250	35.6	192	24.7	95	19.1	171	16.2
Sometimes	278	39.7	338	43.6	158	31.7	292	27.6
Always	173	24.7	246	31.7	245	49.2	594	56.2
Remains sitting								
Rarely	222	31.5	283	36.5	193	38.7	445	42.0
Sometimes	359	51.0	385	49.7	216	43.4	433	40.8
Always	123	17.5	107	13.8	89	17.9	182	17.2
Walks								
Rarely	251	35.8	229	29.5	96	19.3	191	18.0
Sometimes	194	27.6	222	28.6	114	22.9	218	20.5
Always	257	36.6	325	41.9	287	57.8	652	61.5
Lifts/carries/pushes loads								
Rarely	543	77.5	500	64.9	229	46.2	420	39.8
Sometimes	124	17.7	212	27.5	144	29.0	331	31.3
Always	34	4.8	59	7.6	123	24.8	305	28.9
Takes breaks during the work shift								
No	428	61.0	461	60.3	236	47.9	461	43.5
Yes	274	39.0	304	39.7	257	52.1	598	56.5

P_00_ = low physical demand and low psychosocial demand; P_01_ = low physical demand and high psychosocial demand; P_10_ = high physical demand and low psychosocial demand; P_11_ = high physical demand and high psychosocial demand.

The prevalence of MSP among workers exposed to both high physical demand and high psychosocial demand was 38.1% in the LL, 29.8% in the UL, and 46.2% in the back.

Table 3 lists the results of the hierarchical analysis for factors associated with musculoskeletal disorders of the LL. The following variables were associated with MSP in LL: P_10_ - high physical demand and low psychosocial demand (prevalence ratio [PR]: 1.93; 95%CI: 1.46-2.56); P_11_ - high physical demand and high psychosocial demand (PR: 2.36; 95%CI: 1.86-3.00); female sex (PR: 2.49; 95%CI: 1.94-3.21); and working providing direct health care (PR: 1.72; 95%CI: 1.41-2.11). The type of employment category “on contract” was a protective association with MSP in LL (PR: 0.55; 95%CI: 0.45-0.67), as were not participating in leisure activities (PR: 1.33; 95%CI: 1.06-1.67), physical inactivity (PR: 1.50; 95%CI: 1.25-1.79), and “poor” health status (PR: 2.45; 95%CI: 1.58-3.80) (Table 3).

**Table 3:** Prevalence ratios (PR) and 95% confidence intervals (95%CI) for the hierarchical analysis of factors associated with musculoskeletal pain in the lower limbs of health care workers, Feira de Santana, Santo Antônio de Jesus, Jequié, Salvador, Itabuna, and Itaberaba, Brazil, 2011-2012

Variables	Pain in lower limbs				
	Model A PR (95%CI)	Model B PR (95%CI)	Model C PR (95%CI)	Model D PR (95%CI)	Model E PR (95%CI)
Block I					
P^01^	1.13 (0.87-1.45)	1.11 (0.85-1.44)	1.25 (0.87-1.80)	1.11 (0.85-1.45)	1.12 (0.86-1.47)
P^10^	1.98 (1.51-2.58)	1.90 (1.45-2.50)	2.30 (1.56-3.41)	1.85 (1.40-2.44)	1.93 (1.46-2.56)
P^11^	2.50 (1.99-3.13)	2.45 (1.94-3.09)	2.56 (1.84-3.56)	2.44 (1.93-3.10)	2.36 (1.86-3.00)
Block II					
Sex					
Female		2.88 (2.25-3.69)	2.41 (1.73-3.34)	2.59 (1.98-3.39)	2.49 (1.94-3.21)
Age group (years)					
34-43		1.13 (0.91-1.40)			
≥ 44		1.04 (0.82-1.32)			
Marital status					
Married/stable relationship		1.42 (1.14-1.77)	1.12 (0.87-1.45)		
Widowed/divorced/separated/estranged		1.11 (0.80-1.54)	1.10 (0.75-1.62)		
Educational level					
Secondary education/technical college		1.04 (0.67-1.62)			
Higher education		0.80 (0.50-1.26)			
Has children					
Yes		0.97 (0.77-1.23)			
Block IIIa					
Job type					
Direct health care			1.39 (1.08-1.78)	1.72 (1.40-2.10)	1.72 (1.41-2.11)
Time in job (years)					
5-12			1.00 (0.76-1.31)		
≥ 13			0.96 (0.70-1.34)		
Type of employment					
Contract			0.66 (0.51-0.85)	0.58 (0.47-0.70)	0.55 (0.45-0.67)
Work shift					
Day			1.05 (0.51-2.13)		
On call			1.31 (0.60-2.86)		
Working week (hours)					
21-39			0.86 (0.58-1.29)		
≥ 40			0.98 (0.66-1.45)		
Has another job					
Yes			0.90 (0.69-1.19)		
Condition of chairs and tables					
Poor			0.93 (0.71-1.22)		
Technical resources and equipment					
Poor			1.20 (0.90-1.61)		
Level of demand of tasks and resources available					
Poor			1.08 (0.78-1.48)		
Block IIIb					
Is the person responsible for the housework					
Yes				1.15 (0.96-1.38)	
Does the housework					
Yes				1.27 (0.87-1.86)	
Block IV					
Leisure activities					
No		1.46 (1.18-1.82)	1.54 (1.13-2.10)	1.35 (1.08-1.70)	1.33 (1.06-1.67)
Physical activity					
No		1.61 (1.36-1.91)	1.62 (1.27-2.07)	1.56 (1.30-1.87)	1.50 (1.25-1.79)
Self-rated health status					
Poor		2.60 (1.70-3.98)	2.33 (1.26-4.32)	2.57 (1.65-4.01)	2.45 (1.58-3.80)

Model A - Block I = principal independent variable; Model B - Model A + Block II = sociodemographics; Model C - Model B + Block IIIa = Work performed at the unit; Model D - Model C + Block IIIb = Housework; Model E- Model D + Block IV = health status and lifestyle habits; P_01_ = low physical demand and high psychosocial demand; P_10_ = high physical demand and low psychosocial demand; P_11_ = high physical demand and high psychosocial demand.

Variables that were still associated with MSP in UL after the hierarchical analysis were P_10_ - high physical demand and low psychosocial demand (PR: 2.57; 95%CI: 1.79-3.68); P_11_ - high physical demand and high psychosocial demand (PR: 3.42; 95%CI: 2.49-4.70); female sex (PR: 2.87; 95%CI: 1.97-4.17); being the person responsible for the housework (PR: 1.26; 95%CI: 1.00-1.58); doing the housework (PR: 1.75; 95%CI: 1.01-3.05); physical inactivity (PR: 1.27; 95%CI: 1.02-1.59); and “poor” self-rated health status (PR: 4.95; 95%CI: 3.02-8.11) (Table 4).

**Table 4:** Prevalence ratios (PR) and 95% confidence intervals (95%CI) for the hierarchical analysis of factors associated with musculoskeletal pain in upper limbs in health care workers, Feira de Santana, Santo Antônio de Jesus, Jequié, Salvador, Itabuna, and Itaberaba, Brazil, 2011-2012

Variables	Pain in upper limbs				
	Model A PR (95%CI)	Model B PR (95%CI)	Model C PR (95%CI)	Model D PR (95%CI)	Model E PR (95%CI)
Block I					
P^01^	1.12 (0.78-1.61)	1.16 (0.80-1.69)	1.01 (0.59-1.74)	1.14 (0.79-1.66)	1.11 (0.76-1.63)
P^10^	2.65 (1.88-3.73)	2.48 (1.73-3.54)	2.66 (1.59-4.46)	2.43 (1.71-3.47)	2.57 (1.79-3.68)
P^11^	3.79 (2.81-5.11)	3.74 (2.74-5.12)	3.47 (2.22-5.44)	3.61 (2.66-4.92)	3.42 (2.49-4.70)
Block II					
Sex					
Female		3.60 (2.52-5.15)	3.72 (2.25-6.14)	3.14 (2.17-4.54)	2.87 (1.97-4.17)
Age group (years)					
34-43		1.25 (0.96-1.65)	0.99 (0.65-1.49)		
≥ 44		1.56 (1.16-2.10)	1.40 (0.88-2.22)		
Marital status					
Married/stable relationship		1.13 (0.85-1.50)			
Widowed/divorced/separated/estranged		1.04 (0.69-1.58)			
Educational level					
Secondaryeducation/technical college		1.09 (0.64-1.84)			
Higher education		0.91 (0.53-1.58)			
Race/color					
White/yellow		2.35 (0.79-6.94)			
Black/brown		1.76 (0.58-5.36)			
Has children					
Yes		1.40 (1.02-1.90)	1.43 (0.98-2.09)		
Block IIIa					
Job type					
Direct health care			0.84 (0.59-1.18)		
Time in job (years)					
5-12			0.97 (0.67-1.40)		
≥ 13			1.18 (0.73-1.91)		
Type of employment					
Contract			0.73 (0.53-1.01)		
Work shift					
Day			1.08 (0.47-2.51)		
On call			1.01 (0.40-2.52)		
Working week (hours)					
21-39			1.53 (0.88-2.65)		
≥ 40			1.35 (0.79-2.30)		
Has another job					
Yes			0.95 (0.65-1.38)		
Condition of chairs and tables					
Poor			1.01 (0.70-1.44)		
Technical resources and equipment					
Poor			0.89 (0.59-1.32)		
Level of demand of tasks and resources available					
Poor			1.45 (0.94-2.24)		
Block IIIb					
Is the person responsible for the housework					
Yes				1.27 (1.01-1.58)	1.26 (1.00-1.58)
Does the housework					
Yes				1.89 (1.09-3.26)	1.75 (1.01-3.05)
Block IV					
Leisure activities					
No					1.15 (0.87-1.50)
Physical activity					
No					1.27 (1.02-1.59)
Self-rated health status					
Poor					4.95 (3.02-8.11)

Model A - Block I = principal independent variable; Model B - Model A + Block II = sociodemographics; Model C - Model B + Block IIIa = Work performed at the unit; Model D - Model C + Block IIIb = Housework; Model E - Model D + Block IV = health status and lifestyle habits; P_01_ = low physical demand and high psychosocial demand; P_10_ = high physical demand and low psychosocial demand; P_11_ = high physical demand and high psychosocial demand.

The following variables were still associated with back pain after the analysis: P_10_ (PR: 2.13; 95%CI: 1.62-2.81); P_11_ (PR: 2.67; 95%CI: 2.10-3.38); female sex (PR: 1.91; 95%CI: 1.52-2.40); “poor” level of demand of tasks and technical resources available (PR: 1.45; 95%CI: 1.19-1.78); no leisure activities (PR: 1.34; 95%CI: 1.06-1.68); no physical activity (PR: 1.30; 95%CI: 1.09-1.55); and “poor” self-rated health status (PR: 3.44; 95%CI: 2.07-5.71); type of employment “on contract” was associated, but as a protective factor (PR: 0.66; 95%CI: 0.54-0.79) (Table 5).

**Table 5:** Prevalence ratios (PR) and 95% confidence intervals (95%CI) for the hierarchical analysis of factors associated with musculoskeletal back pain in health care workers, Feira de Santana, Santo Antônio de Jesus, Jequié, Salvador, Itabuna, and Itaberaba, Brazil, 2011-2012

Variables	Back pain				
	Model A PR (95%CI)	Model B PR (95%CI)	Model C PR (95%CI)	Model D PR (95%CI)	Model E PR (95%CI)
Block I					
P^01^	1.33 (1.03-1.71)	1.37 (1.05-1.78)	1.68 (1.17-2.42)	1.21 (0.92-1.58)	1.26 (0.97-1.65)
P^10^	2.25 (1.72-2.93)	2.30 (1.74-3.03)	2.47 (1.66-3.66)	2.08 (1.58-2.75)	2.13 (1.62-2.81)
P^11^	2.96 (2.36-3.71)	2.99 (2.36-3.79)	3.15 (2.26-4.40)	2.68 (2.12-3.40)	2.67 (2.10-3.38)
Block II					
Sex					
Female		2.12 (1.69-2.66)	2.25 (1.66-3.07)	2.01 (1.58-2.56)	1.91 (1.52-2.40)
Age group (years)					
34-43		0.87 (0.70-1.08)			
≥ 44		0.81 (0.63-1.03)			
Marital status					
Married/stable relationship		1.21 (0.97-1.52)			
Widowed/divorced/separated/estranged		0.97 (0.69-1.37)			
Educational level					
Secondary education/technical college		1.49 (0.92-2.40)			
Higher education		1.55 (0.95-2.53)			
Race/color					
White/yellow		2.15 (0.96-4.83)			
Black/brown		1.95 (0.85-4.46)			
Has children					
Yes		1.24 (0.98-1.57)			
Block IIIa					
Job type					
Direct health care			1.21 (0.94-1.56)		
Time in job (years)					
5-12			0.97 (0.74-1.26)		
≥ 13			1.04 (0.75-1.45)		
Type of employment					
Contract			0.72 (0.56-0.93)	0.64 (0.53-0.77)	0.66 (0.54-0.79)
Work shift					
Day			0.82 (0.43-1.55)		
On call			0.94 (0.47-1.90)		
Working week (hours)					
21-39			1.02 (0.69-1.51)		
≥ 40			0.98 (0.67-1.42)		
Has another job					
Yes			1.22 (0.93-1.60)		
Condition of chairs and tables					
Poor			1.15 (0.88-1.52)		
Technical resources and equipment					
Poor			1.15 (0.85-1.55)		
Level of demand of tasks and resources available					
Poor			1.40 (1.01-1.94)	1.52 (1.24-1.86)	1.45 (1.19-1.78)
Block IIIb					
Is the person responsible for the housework					
Yes				0.92 (0.76-1.10)	
Does the housework					
Yes				1.37 (0.96-1.96)	
Block IV					
Leisure activities					
No					1.34 (1.06-1.68)
Physical activity					
No					1.30 (1.09-1.55)
Self-rated health status					
Poor					3.44 (2.07-5.71)

Model A - Block I = principal independent variable; Model B - Model A + Block II = sociodemographics; Model C - Model B + Block IIIa = Work performed at the unit; Model D - Model C + Block IIIb = Housework; Model E - Model D + Block IV = health status and lifestyle habits; P_01_ = low physical demand and high psychosocial demand; P_10_ = high physical demand and low psychosocial demand; P_11_ = high physical demand and high psychosocial demand.

## DISCUSSION

The present study investigated associations between physical and psychosocial demands and occurrence of MSP in health care workers using hierarchical analysis. The following factors were associated with MSP in all three body areas assessed: female sex, lack of physical activity, and “poor” self-rated health status. Being a contract worker was a protective factor for MSP in the LL and back.

It was observed that high percentages of workers were exposed to physical and psychosocial demands. These factors were associated with musculoskeletal disorders.^18,19^ Although this study was restricted to health care workers, this finding is similar to findings from research conducted with other categories of workers in Brazil^4,20-22^ and worldwide.^7^

Absence of leisure activity was associated with MSP in two of the body areas studied. Exposure to physical demands at work may affect leisure activities and physical activities,^22^ since, for many workers, limited free time outside of work may mean that they use their available free time to rest and recover from the physical effort they have exerted at work,^20^ contributing to reduce their engagement in these practices (even though they could function to promote mental and physical health).

In line with the results of the present study, Barbosa et al.^10^ also found associations between a lack of physical activity and musculoskeletal complaints in health care workers. Resistance and muscle strength acquired during physical activity protect workers from MSP and reduce its impacts on health. This is irrespective of the conditions in which physical work is performed.^20^

“Poor” self-rated health status was associated with MSP. The high prevalence rates and powerful associations between these variables may reflect a preexisting MSP condition, considering that this is an influence on living conditions at work and capable of affecting the worker’s opinion.

Direct provision of health care was another variable that was associated with MSP, which confirms the findings of other investigations that show that the job categories dentist, dental auxiliary, and community health worker were also associated with this condition.^10^ The physical and psychological organizational demands to which health professionals are subjected contribute to this situation.^23^ Repetition of movements and remaining in a static position for long periods while working increase risk of MSP,^24^ because they cause compression of musculoskeletal structures. Repetition and static positions are both characteristic of the activities performed by health care professionals.^25^

The findings of the present study demonstrate that workers who are on contracts had lower prevalence of MSP in the LL and back. According to published data, better health status would traditionally be expected in people with stable employment. It is therefore necessary to analyze this unexpected study result in greater detail. One likely explanation is related to the profile of health care employment. Recently, employment of more qualified workers (professionals with higher education qualifications in medicine, nursing, and dentistry) has been contracted on a temporary basis in Brazil, in particular because the standard public competitions for vacancies have not been held. In contrast, other occupations at the middle levels, such community health workers, are subject to legislation that makes public competitions mandatory. As a result, the advantages of a permanent job may not be sufficient to guarantee working conditions with less exposure. Additionally, it is also possible that workers with permanent jobs do not perform roles involving as much direct health care provision (which was one of the variables associated with pain in the body areas studied) as other jobs. It is therefore plausible to suppose that they may be less exposed to high physical and psychosocial demands. However, the prevalence rates of MSP in contract workers were not very different between those with jobs that do and do not provide direct health care, at 20.6 and 17.2% in LL and 28.1 and 24.3% in the back, respectively, although they were higher in those who do provide direct care.

Female sex was associated with MSP in all three areas of the body studied, confirming other studies that have investigated the subject.^9,10,22,26,27^ Factors such as a double work load (which reduces the time available for physical activity or relaxation, thus limiting activities that protect from injuries and help to prevent them^10,28^) and the anthropometric differences between the sexes and between the muscle fibers of men and women (who are weaker and less resistant) can also contribute to emergence of musculoskeletal injuries.^29^

The double work load of women is clear from findings observed over the course of this study. Majorities of them do the housework and/or are responsible for the housework, are married, and have children. As such, the differences between the sexes should be taken into account in fair division of tasks in the home, bearing in mind the double work load put on women, and their physiological characteristics.^22^

Other important factors that could contribute to this finding, particularly in relationship to spinal complaints, are pregnancy and postpartum, since the changes to the body and hormones that occur during pregnancy lead to greater flexibility in the spine and hips, causing changes to these structures.^9^

Doing the housework and being the principal person responsible for it, which are both factors that contribute to emergence of painful symptomology in women, were both associated with back pain. These findings show that women who reported a painful complaint in this region were also responsible for doing the housework (93.3%).

The present study is subject to limitations. One of these is the cross-sectional study design, which cannot establish cause and effect relationships, suggesting a need for longitudinal studies of the subject. The healthy worker bias should also be acknowledged, since workers who were off sick or had left the profession did not take part in the study.

## CONCLUSIONS

High prevalence rates of exposure to physical and psychosocial demands were observed in the present study. These were associated with MSP in the LL, in the UL, and in the back in analyses with the hierarchical model, with a particular emphasis on the associations when both exposures and high physical demand were present, reinforcing the hypothesis that these characteristics are important factors for occurrence of the outcome.

The results of the hierarchical analysis show the importance of considering the multiple exposures to which workers are exposed and the relationships that are established between the different exposure factors, offering more in-depth understanding and the chance to seek measures that could reduce the impacts of the physical and psychosocial demands of work and, consequently, the rate of MSP in workers. Such measures could be implemented in the form of actions that lead to restructuring of organizational aspects, minimization of work demands, and improvement of working conditions. Additionally, the associations between female sex and MSP reveal a need to reflect on strategies for prevention in this group.
